# Evaluation of foliar fungus‐mediated interactions with below and aboveground enemies of the invasive plant *Ageratina adenophora*

**DOI:** 10.1002/ece3.7072

**Published:** 2020-11-25

**Authors:** Kai Fang, Li‐Min Chen, Han‐Bo Zhang

**Affiliations:** ^1^ School of Ecology and Environmental Science Yunnan University Kunming China; ^2^ State Key Laboratory for Conservation and Utilization of Bio‐Resources in Yunnan Yunnan University Kunming China; ^3^ Sichuan Academy of Grassland Sciences Chengdu China

**Keywords:** foliar fungi, habitat, herbivorous insect, invasive plant, pathogens

## Abstract

Plant–fungal associations are frequently key drivers of plant invasion success. Foliar fungi can benefit their invasive hosts by enhancing growth promotion, disease resistance and environmental stress tolerance. However, the roles of foliar fungi may vary when a given invasive plant faces different stresses. In this study, we designed three independent experiments to evaluate the effects of a foliar fungus, *Colletotrichum* sp., on the growth performance of the invasive plant *Ageratina adenophora* under different soil conditions, as well as the responses of *A. adenophora* to the foliar fungal pathogen *Diaporthe helianthi* and to herbivory. We found that the soil type was the most influential factor for the growth of *A. adenophora*. The role of the foliar fungus *Colletotrichum* sp. varied in the different soil types but generally adversely affected leaf development in *A. adenophora*. *Colletotrichum* sp. may be a weak latent foliar pathogen that can enhance the pathogenicity of *D. helianthi* on leaves of *A. adenophora* and marginally reduce signs of herbivory by natural insects in the wild on *A. adenophora* seedlings. In general, the benefits of the foliar fungus *Colletotrichum* to the fitness of *A. adenophora* are not significant in the context of this experimental design. However, our data highlight the need to consider both aboveground and belowground biota in different soil habitats when evaluating the effects of foliar fungi.

## Graphical abstract

Soil type played the most important role in the growth of *A. adenophora*, and the role of the foliar fungus *Colletotrichum* sp. varied according to different habitats but in general adversely affected the growth of *A. adenophora*. The *Colletotrichum* sp. might be a weak latent pathogen and enhanced the pathogenicity of *Diaporthe helianthi* on leaves of *A. adenophora*. *Colletotrichum* sp. marginally reduced the feeding frequency of seedlings by natural enemies in the wild.

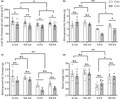

## 1 INTRODUCTION

The rapid expansion of exotic plants has caused serious damage to the structure and function of invaded ecosystems and has resulted in enormous social and economic losses (Richardson & Ricciardi, 2013; Vilà et al., 2010). Determining why invasive plants succeed in their introduced range has been a major goal of invasion ecology. Plant–fungal associations are frequently key drivers of plant invasion success; a fungal species can act as a mutualist by enhancing plant defense, growth and stress tolerance or as a pathogen to cause establishment failure (Dickie et al., 2017; Flory & Clay, 2013). Foliar fungi are diverse in terrestrial ecosystems, including endophytes and epiphytes, and are often assumed to be mutualists, latent saprotrophs or pathogens of plants (Arnold, 2007; Osono et al., 2004; Schulz et al., 1999). Foliar fungi have also been observed to be capable of altering plant disease severity (Arnold et al., 2003; Busby et al., 2016). For exotic plants, foliar fungi can assist the host to grow, resist disease, and change herbivory. For example, Aschehoug et al. (2012) indicated that the inoculation of endophytic *Alternaria* (CID120) on the invasive forb *Centaurea stoebe* could directly increase its growth. Currie et al. (2019) demonstrated that foliar fungi (*Colletotrichum acutatum*, *Alternaria alternata* and *Cladosporium oxysporum*) appeared to mitigate the effect of the pathogen *Puccinia komarovii* on the invasive weed *Impatiens glandulifera*. Rudgers and Clay (2008) showed that the foliar fungus *Neotyphodium coenophialum* could significantly reduce the abundance and diversity of herbivorous arthropods on the invasive plant *Lolium arundinaceum*.

In addition to aboveground biota, invasive plants also interact with belowground biota. Escape from soil‐specific host pathogens has been suggested to play a key role in determining the success of exotic plant invasion into local ecosystems, and this phenomenon is referred to as positive plant–soil feedback (PSF) (Callaway et al., 2004; Hawkes et al., 2005; Klironomos, 2002; Kulmatiski et al., 2008). Nonetheless, exotic plants have not experienced uniformly positive PSF, for example, Andonian et al. (2011) indicated that an invasive weed experienced negative PSF; Crawford and Knight (2017) found that *Lespedeza cuneata* experienced positive PSF in invaded soils only in the absence of competition; and for some plant species, a positive PSF was observed in a glasshouse but not in the field ((Schittko et al., 2016). These discrepancies suggest that the PSF experienced by the exotic invaders is context dependent and may involve the soil habitat type in which the plants grow (Pizano et al., 2019).

More interestingly, interactions of belowground and aboveground biota may exist when performing PSF studies. For example, the invasive plant *I. glandulifera* experienced a positive PSF, and the effect of the PSF even extended beyond the soil microbial community to affect foliar fungi, which in turn enhanced resistance to herbivory in *I. glandulifera* and thus accentuated the invasive properties of this species (Pattison et al., 2016). In reality, invasive hosts expand into different habitats across a large geographical scale (Vilà et al., 2010) and potentially interact with diverse belowground biota (e.g., soilborne pathogens) and aboveground biota (e.g., foliar fungi). However, it is unclear whether the invasive host experiences distinct soil biota effects in different habitats and how the roles of the soil biota change after a foliar fungus is introduced; in addition, it is also unknown whether a foliar fungus can modify both aboveground diseases and herbivory in the invasive host.

The invasive plant *Ageratina adenophora* (Sprengel) R. M. King and H. Robinson is a perennial herb of the Compositae family that is native to Central America. The spread of *A. adenophora* is considered to be a severe problem in more than 30 countries in Asia, Africa, Oceania, Europe and North America (Datta et al., 2019). In China, this weed has been reported to reduce the diversity of native plant species, crop production in agricultural land and forage production in pastures; moreover, this weed is poisonous to domestic animals (Poudel et al., 2019). Therefore, understanding the mechanisms of successful weed invasion is ecologically and economically important. Previous results have indicated that the roles of soil biota in the establishment of *A. adenophora* are habitat dependent, for example, Niu et al. (2007) indicated that soil biota collected from mixed evergreen‐broadleaf‐deciduous forests had strong inhibitory effects on *A. adenophora* growth; however, Xiao et al. (2014) indicated that sterilization had no significant effect on *A. adenophora* biomass growing in soils collected from a tropical botanical garden. Nevertheless, once established, *A. adenophora* can change the soil biota to promote its own growth (Niu et al., 2007), for example, by increasing the abundance of nitrogen‐fixing bacteria (Xu et al., 2012) and root endophytic *Enterobacter* bacteria (Chen et al., 2019). Moreover, *A. adenophora* has also been reported to host various foliar fungi, including pathogens (Poudel et al., 2019; Zhou et al., 2010) and endophytes (Mei et al., 2014, Fang et al., 2019). In addition, foliar herbivory was frequently observed in our routine investigations. These data suggest the occurrence of potential aboveground and belowground biotic interactions with *A. adenophora*, which remain to be characterized. In this study, we designed three independent experiments to determine the effect of a foliar fungus, *Colletotrichum,* on the growth performance of *A. adenophora* in different soil conditions, as well as the functional responses of *A. adenophora* to aboveground pathogenic fungi and herbivores.

## 2 MATERIALS AND METHODS

### 2.1 Cultivating seedlings of *A. adenophora*

Seeds were collected from wild populations of *A. adenophora* in Kunming city (24°58′22″ N, 102°27′49″ E, 1980 m), Yunnan Province, Southwest China. Completely filled seeds were selected and surface sterilized by submerging them in 95% ethanol for 30 s and 2% sodium hypochlorite for 20 min and rinsing them with sterile water three times. After surface sterilization, the seeds were submerged in sterile water for 24 hr and then planted in pots containing sterile soils. The soils used to grow the plants were sterilized three times at 24‐hr intervals by autoclaving (121°C, 0.135 MPa, 2 hr). Pots were disinfected by submerging them in 0.2 g/L potassium permanganate solution for 30 min. Plants were cultivated in an RXZ‐380D growth chamber (Ningbo Southeast Instrument Co., Ltd, Ningbo, China) with a temperature of 25 ± 1°C, light intensity of 80–100 µmolm^−2^ s^−1^, 12‐hr photoperiod and humidity of 80 ± 5%. The germinated seedlings were supplemented with the same amount of sterile water in each pot every day as needed and watered with Hoagland nutrient solution (detailed in Table 1) once a month. The seedlings were grown for 5 months, and then those of similar sizes (approximately 20 cm shoot length) were randomly selected for the subsequent experiments.

**Table 1:** Formulation of Hoagland nutrition solution

Nutriment		mM	g/L
Micronutrients	Ca(NO_3_)·4H_2_O	4.0	0.94
	MgSO_4_·7H_2_O	2.0	0.52
	KNO_3_	6.0	0.66
	NH_4_H_2_PO_4_	1.0	0.12
	Chelate iron	–	0.07
Large nutrients	H_3_BO_3_		28
	MnSO_4_·H_2_O		34
	CuSO_4_·5H_2_O		1.0
	ZnSO_4_·7H_2_O		2.2
	(NH_4_)_6_MO_7_O_24_·4H_2_O		1.0
	H_2_SO_4_		5.0 ml

Note

Hoagland nutrient solution is a mixture of 0.1 ml of micronutrients and 1 L of large nutrients, and the pH is adjusted to 6.7.

### 2.2 Inoculation and detection of foliar fungi

The fungus JK99 was previously isolated from healthy leaves of *A. adenophora*, making up nearly 30% of the total isolated fungi (Mei et al., 2014). It has been identified as *Colletotrichum* sp. (see colony morphology in Figure S1. Its ITS gene was sequenced and was completely identical to the sequence of a strain of *Colletotrichum* sp. A285 that we previously submitted to GenBank with an accession number of MK247540). Calcium carbonate medium (1,000 ml of distilled water containing 30 g of CaCO_3_, 20 g of glucose and 18 g of agar) was used for sporulation at 28°C. The preparation of the spore suspension and inoculation were performed as previously reported (Arnold et al., 2003). Briefly, the spores produced on the plate were washed with 0.5% sterilized gel solution (100 ml of distilled water containing 0.5 g of gel). The mycelium was removed with sterile filter paper, and the spore suspension was diluted to 10^6^ CFU/ml using a haemocytometer. Then, the *A. adenophora* leaves were misted to saturation on both the upper and lower surfaces using this spore suspension with a sterile sprayer, and the control group was sprayed with sterile 0.5% gel solution. The whole plant was wrapped in a sterile black plastic bag for 48 hr to promote fungal infection, and the fungal infection was detected after 15 days, according to Arnold et al. (2003). The leaves inoculated and not inoculated (control plants) with *Colletotrichum* sp. (named Col+ and Col−, respectively) were surface sterilized by submerging in 0.5% sodium hypochlorite for 2 min and 70% ethanol for 2 min and rinsing with sterile water 3 times, and then the surface‐sterilized leaves were cut into fragments of approximately 6 mm^2^. Sixteen fragments were randomly selected from each leaf sample, placed on a malt extract agar (MEA, 1,000 ml of distilled water containing 30 g of malt extract, 3 g of soy peptone and 18 g of agar; pH 5.6 ± 0.2) plate, sealed with parafilm and cultured at room temperature for several days (Figure S2). The number of leaf fragments with fungi and the colony morphology were observed and recorded. The fungal infection rate of the leaves (Col+ and Col−) was calculated by dividing the number of fragments with fungi by the total number of fragments.

### 2.3 Growth experiments with *A. adenophora* in different soil types

The soils used in this experiment were collected from agricultural land and a forest in Kunming city, Yunnan Province, Southwest China, and are referred to as agricultural soil (AS) and forest soil (FS). The agricultural soil collection site commonly rotates maize, pea and cabbage crops. The forest soil collection site is dominated by *Pinus yunnanensis* and *Cyclobalanopsis glaucoides*. Both sites have red soil. The soil properties are shown in Table 2.

**Table 2:** Chemical composition of soil

Soil type	pH	Organic matter	Total N (%)	Total P (%)	Total K (%)	Available N (mg/kg)	Available P (mg/kg)	Available K (mg/kg)
Agriculture soil	6.41 ± 0.07	4.09 ± 0.17	0.20 ± 0.01	0.19 ± 0.01	0.42 ± 0.02	183.49 ± 8.47	33.47 ± 3.36	370.53 ± 19.92
Forest soil	7.56 ± 0.09	9.42 ± 0.56	0.45 ± 0.03	0.22 ± 0.01	0.90 ± 0.16	315.15 ± 18.65	44.02 ± 7.01	430.61 ± 19.94
*P−*value	0.004	0.004	0.004	0.020	0.004	0.004	0.025	0.004

Note

Nonparametric Mann–Whitney U tests were used to identify the differences in soil chemical composition between agriculture soil and forest soil. Each soil type contains six replicates.

**Figure 1:**
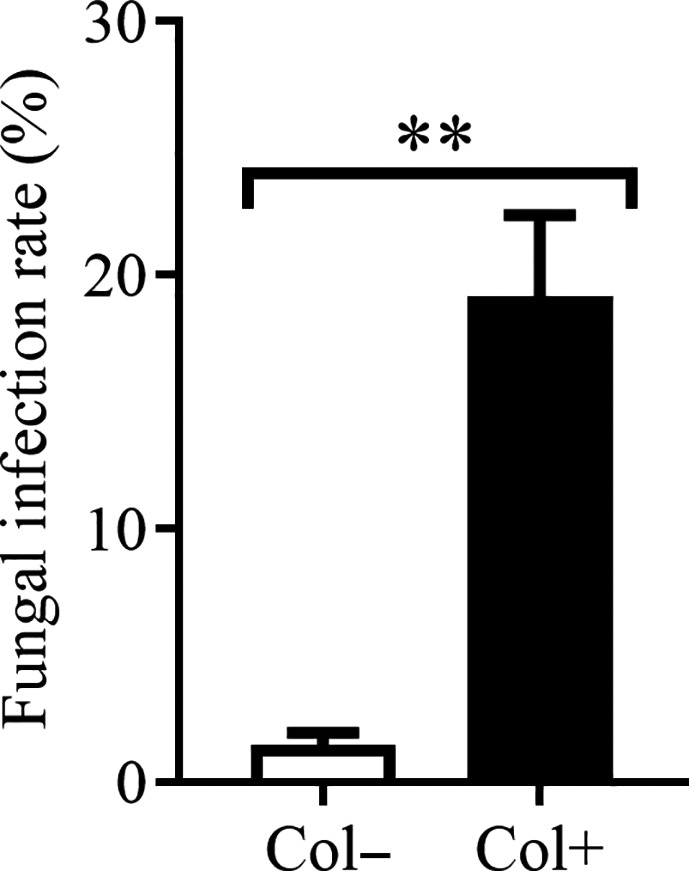
The infection rate of foliar fungus *Colletotrichum* sp. in growth experiment. Col+ and Col− represent seedlings with and without *Colletotrichum* sp., respectively. Nonparametric Mann–Whitney U tests were used to identify the difference in fungal infection rate between leaves with and without the foliar fungus *Colletotrichum* sp. (** represents *p* < 0.01). The error bar represents the standard error

A total of 8 treatments were used to evaluate the effects of the different soil conditions on the growth of *A. adenophora* seedlings, that is, 2 soil types (AS, FS) × 2 treatments (soil sterilization, nonsterilization) × 2 inoculations (with, without *Colletotrichum* sp.) (Figure S3). Because we focused on the role of soil microbes in the growth of the host plant, the soil as an inoculation source was mixed with a sterilized matrix at a volume ratio of 1:9 to lessen the nutrient effects of the different soil types (Whitaker et al., 2017). The sterilized matrix was a mixture of equal proportions of agricultural soil and forest soil, and high‐pressure steam sterilization was carried out as described above. Then, 5‐month‐old *A. adenophora* seedlings (Col+ and Col−) were transplanted into sterilized pots with the different soil treatments. The plants were cultured in an RXZ‐380D growth chamber (Ningbo Southeast Instrument Co., Ltd, Ningbo, China) at a temperature of 25 ± 1°C, light intensity of 80–100 µmolm^−2^s^−1^, 12‐hr photoperiod and humidity of 80 ± 5% and watered with the same amount of sterile water as needed every day. After 2 months, the leaf dry biomass (LDB) per unit area, aboveground dry biomass (ADB), belowground dry biomass (BDB) and shoot length (SL) were measured. Six seedlings (replicates) were planted in each treated soil, and three of the seedlings were randomly selected for fungal infection rate (FIR) detection. The FIR was significantly different between the seedlings with and without *Colletotrichum* sp. inoculation (Figure 1, FIR(Col−) = 1.46%, FIR(Col+) = 19.17%). Since half of the seedlings were used to measure the fungal infection rate, the biomass data (ADB, BDB) included only the remaining three replicates in each treatment, and the SL measurements had 6 replicates. The LDB per unit area was measured by punching the first five pairs of mature leaves with a 5‐mm inner diameter punch to obtain leaf disks and converting them to leaf weight per square meter after weighing. There were 3 replicates for each leaf age and 15 replicates for each treatment. Dry biomass was measured by drying the plant samples at 65°C for 48 hr.

### 2.4 Inoculation experiment with leaf pathogenic fungi

**Figure 2:**
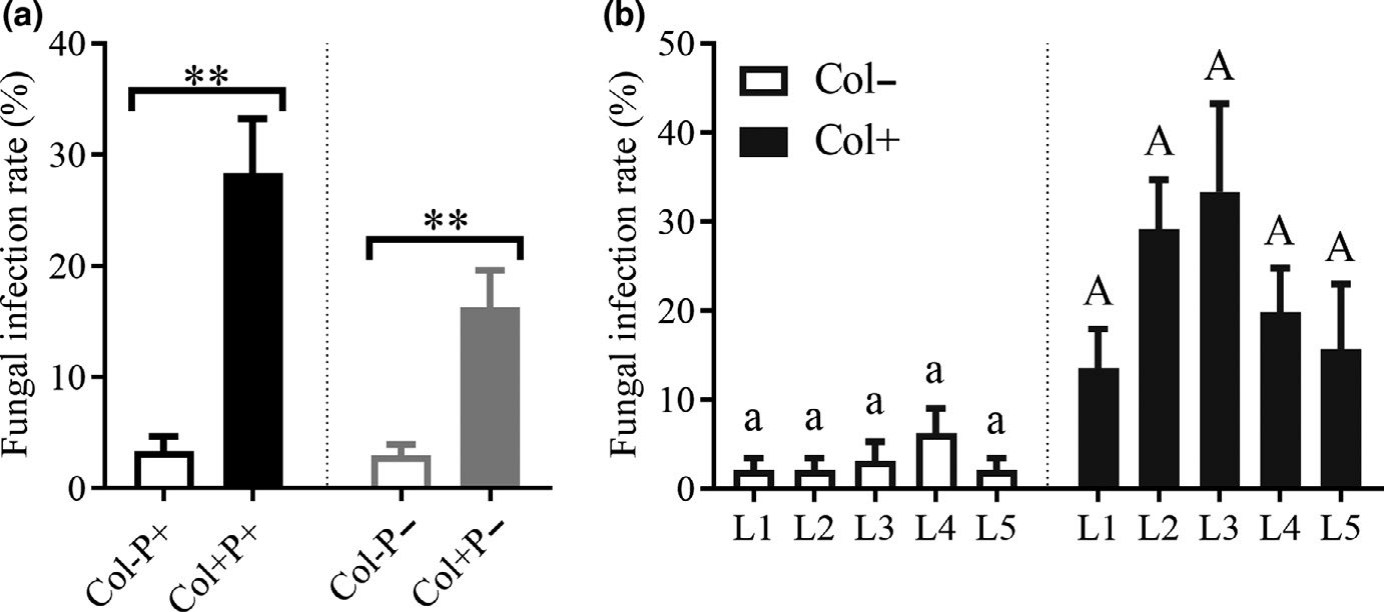
The infection rate of foliar fungus *Colletotrichum* sp. in pathogen experiment. Col+ and Col− represent seedlings with and without *Colletotrichum* sp., respectively (a, b). P+ and P− represent with and without *D. helianthi*, respectively (a). L1‐5 represent different leaf ages (b). Nonparametric Mann–Whitney U tests were used to identify the difference in fungal infection rate between leaves with and without *Colletotrichum* sp. The fungal infection rate was significantly different between Col+ and Col− leaves (a) (** represents *p* < 0.01), but was not significant between different leaf ages, where the same letter means that there was no significant difference between different ages of leaves either with (uppercase letter) or without (lowercase letter) *Colletotrichum* sp. (b). The error bar represents the standard error

The pathogenic fungus used in this experiment was previously isolated from spots on *A. adenophora* leaves, and a phylogenetic analysis of the ITS gene indicated that it was close to *Diaporthe helianthi* (JK58) (see colony morphology in Figure S4, with GenBank accession No. JN854227 for the ITS gene). Its pathogenicity (Figure S5) was verified by the method previously described by Gilbert and Webb (2007). Briefly, the pathogenic fungi cultured on potato dextrose agar (PDA, 1,000 ml of distilled water containing 200 g of potato, 10 g of dextrose and 18 g of agar) medium were made into agar disks with a 3‐mm internal diameter punch. A sterile needle was used to lightly wound the underside of the leaf. The agar disk was then attached to the wound and secured with a hairpin. The control group was inoculated with sterile agar disks. The spot area was measured on day 7 after inoculation and then again every 7 days 4 consecutive times. The inoculation experiments consisted of 4 treatments, called Col−P− (no *Colletotrichum*, no pathogen), Col−P+ (no *Colletotrichum*, pathogen), Col+ P− (*Colletotrichum*, no pathogen) and Col+ P+ (*Colletotrichum*, pathogen). The first five pairs of mature leaves of the seedlings, representing five leaf ages, were inoculated with pathogens, and 9 replicates were inoculated for each leaf age for each treatment. A total of 180 inoculations were carried out. The FIR of *Colletotrichum* sp. was significantly different between Col+ and Col− seedlings (Figure 2a, FIR(Col−P+) = 3.33%, FIR(Col+ P+) = 28.33%, FIR(Col−P−) = 2.92%, FIR(Col+ P−) = 16.30%), but there was no difference in FIR between leaf ages (L1‐5) (Figure 2b, FIR(Col−): L1 = 2.08%, L2 = 2.08%, L3 = 3.13%, L4 = 6.25%, L5 = 2.08%; FIR(Col+): L1 = 13.54%, L2 = 29.17%, L3 = 33.33%, L4 = 19.79%, L5 = 15.63%).

### 2.5 Herbivory experiment

**Figure 3:**
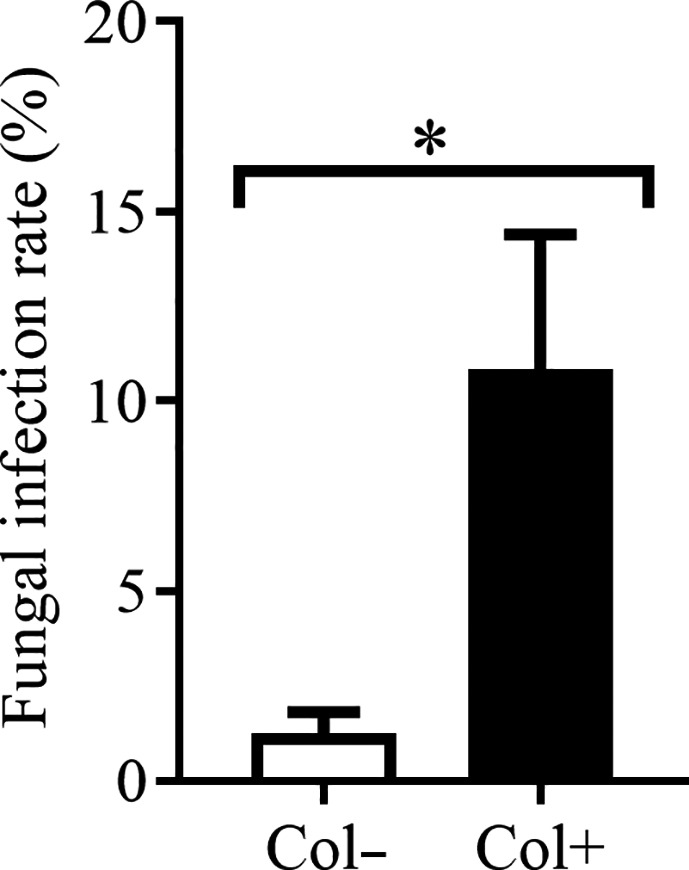
The infection rate of foliar fungus *Colletotrichum* sp. in herbivory experiment. Col+ and Col− represent seedlings with and without *Colletotrichum* sp., respectively. Nonparametric Mann–Whitney U tests were used to identify the difference in fungal infection rate between leaves with and without *Colletotrichum* sp. (* represents *p* < 0.05). The error bar represents the standard error

To date, no report has characterized the aboveground insect enemies of *A. adenophora*, except that a native gallfly of Mexico, *Procecidochares utilis* Stone, has been introduced to control *A. adenophora* (Poudel et al., 2019). However, foliar herbivory is frequently observed in our routine investigations. Thus, we performed herbivory experiments in the wild. Briefly, Col+ and Col− seedlings cultured in the laboratory (the FIR was significantly different, see Figure 3, FIR(Col−) = 1.25%, FIR(Col+) = 10.83%) were placed at random and half a meter apart in a wild plant community in Xishan Forest Park, Kunming (24°58′22″ N, 102°27′49″ E, 1980 m), Yunnan Province. After a week in the field, the seedlings were brought back to the laboratory to record feeding by a guild of natural herbivores in the wild. In most *A. adenophora* individuals, only rare herbivory occurred on very few leaves during the period of our experiment; thus, we only evaluated signs of herbivory from insects attracted to *A. adenophora*. We used “1” to represent the occurrence of feeding when one individual of *A. adenophora* was observed to show herbivory, regardless of the amount of herbivory damage, and “0” to represent that no feeding occurred. The signs of herbivory were calculated and compared between Col+ and Col− seedlings. Each treatment contained 14 individuals, for a total of 28 seedlings.

### 2.6 Data analysis

Nonparametric Mann–Whitney U tests were used to identify the differences in growth between inoculated and noninoculated seedlings, sterilized and nonsterilized soil and agricultural and forest soil, as well as differences in the fungal infection rate, leaf spot area and seedling feeding frequency in the wild between Col+ and Col− leaves. Linear regression was used to analyze the relationships between leaf spot area and leaf age as well as infection time.

All analyses were performed using SPSS, version 22.0 (SPSS Inc., Chicago, IL, USA). Visualizations of the fungal infection rate, growth performance, leaf spot area and feeding frequency were generated using GraphPad Prism 7 (GraphPad Software Inc., San Diego, CA, USA).

## 3 RESULTS

### 3.1 Effects of the foliar fungus *Colletotrichum* sp. on the growth of *A. adenophora* depending on the soil type

**Figure 4:**
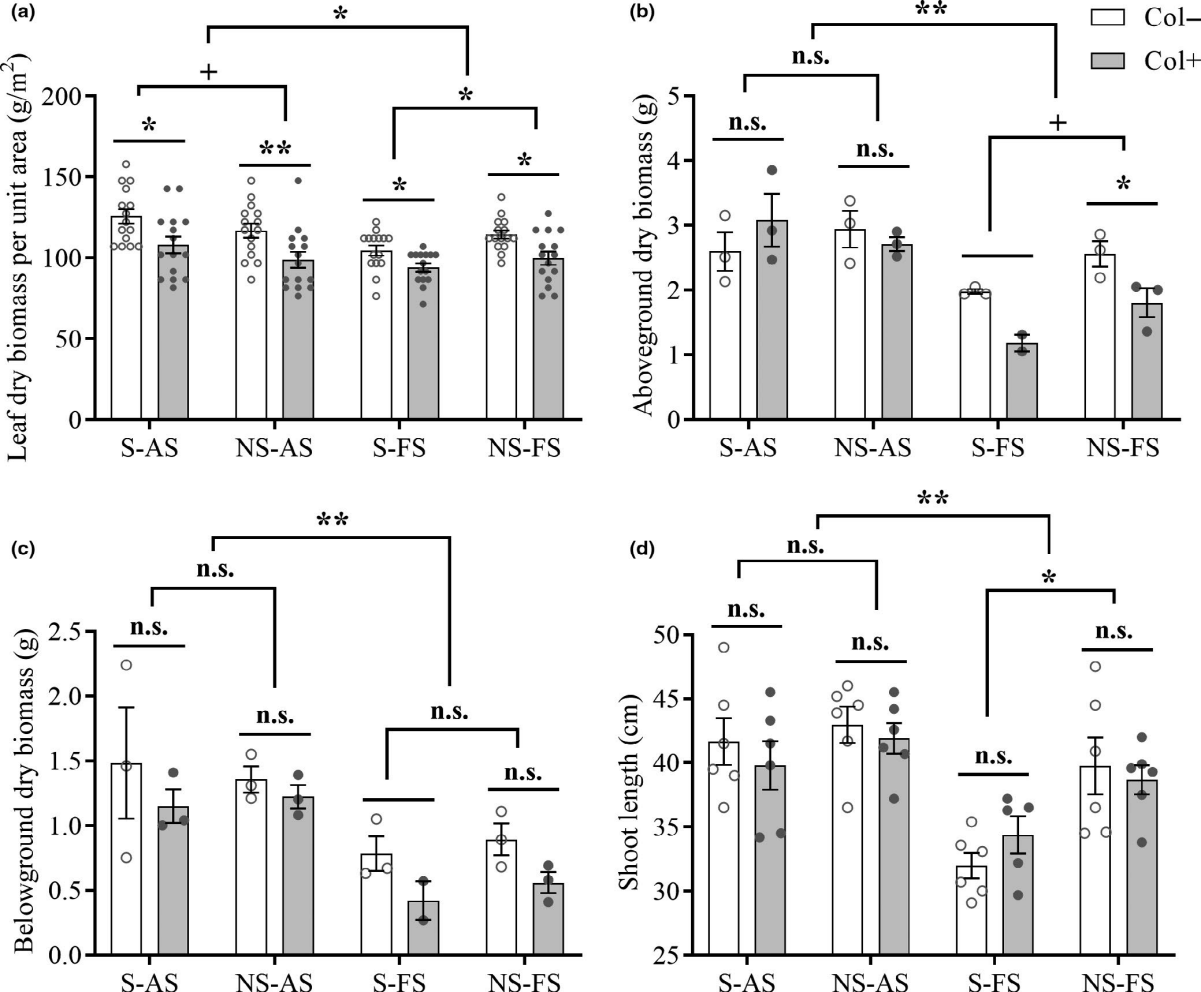
Effects of the foliar fungus *Colletotrichum* sp. on the growth of *A. adenophora* in different soil conditions. Col+ and Col− represent seedlings with and without *Colletotrichum* sp., respectively; AS and FS represent agricultural and forest soil, respectively; S and NS represent sterilization and nonsterilization, respectively. Nonparametric analysis was used to compare the differences in growth performance between Col+ and Col− seedlings, sterilized and nonsterilized soils and agricultural and forest soils (n.s. represents *p* > 0.10, + represents *p* < 0.10, * represents *p* < 0.05, ** represents *p* < 0.01). The error bar represents the standard error. Since one Col+ seedling died in the S‐FS treatment, statistical analysis between the Col+ and Col− seedlings was not performed for the biomass measurements (b, c)

The plants grew better in the agricultural soil (AS) than in the forest soil (FS) (Figure 4, AS versus FS, *p*_(a)_ = 0.014, *p*_(b)_ < 0.001, *p*_(c)_ < 0.001, *p*_(d)_ < 0.001). Inoculation with *Colletotrichum* sp. significantly decreased the leaf dry biomass (LDB) per unit area in both agricultural and forest soil (Figure 4a, Col+ versus Col−, *p*_(S‐AS)_ = 0.019, *p*_(NS‐AS)_ = 0.008, *p*_(S‐FS)_ = 0.017, *p*_(NS‐FS)_ = 0.013); the inoculation also decreased aboveground dry biomass (ADB), but only in nonsterilized forest soil (Figure 4b, Col+ versus Col−, *p*_(NS‐FS)_ = 0.0495). Sterilization improved the LDB per unit area in agricultural soil (Figure 4a, sterilization (S) versus nonsterilization (NS), *p*_(AS)_ = 0.0996) but decreased the LDB per unit area (Figure 4a, S versus NS, *p*_(FS)_ = 0.023), ADB (Figure 4b, S versus NS, *p*_(FS)_ = 0.054) and shoot length (SL) (Figure 4d, S versus NS, *p*_(FS)_ = 0.001) in forest soil. No impact of sterilization was observed on belowground dry biomass (BDB) in either type of soil (Figure 4c, S versus NS, *p*_(AS)_ = 0.749, *p*_(FS)_ = 0.361).

### 3.2 Enhancing the pathogenicity of *D. helianthi* on *A. adenophora* leaves through inoculation with the foliar fungus *Colletotrichum* sp

**Figure 5:**
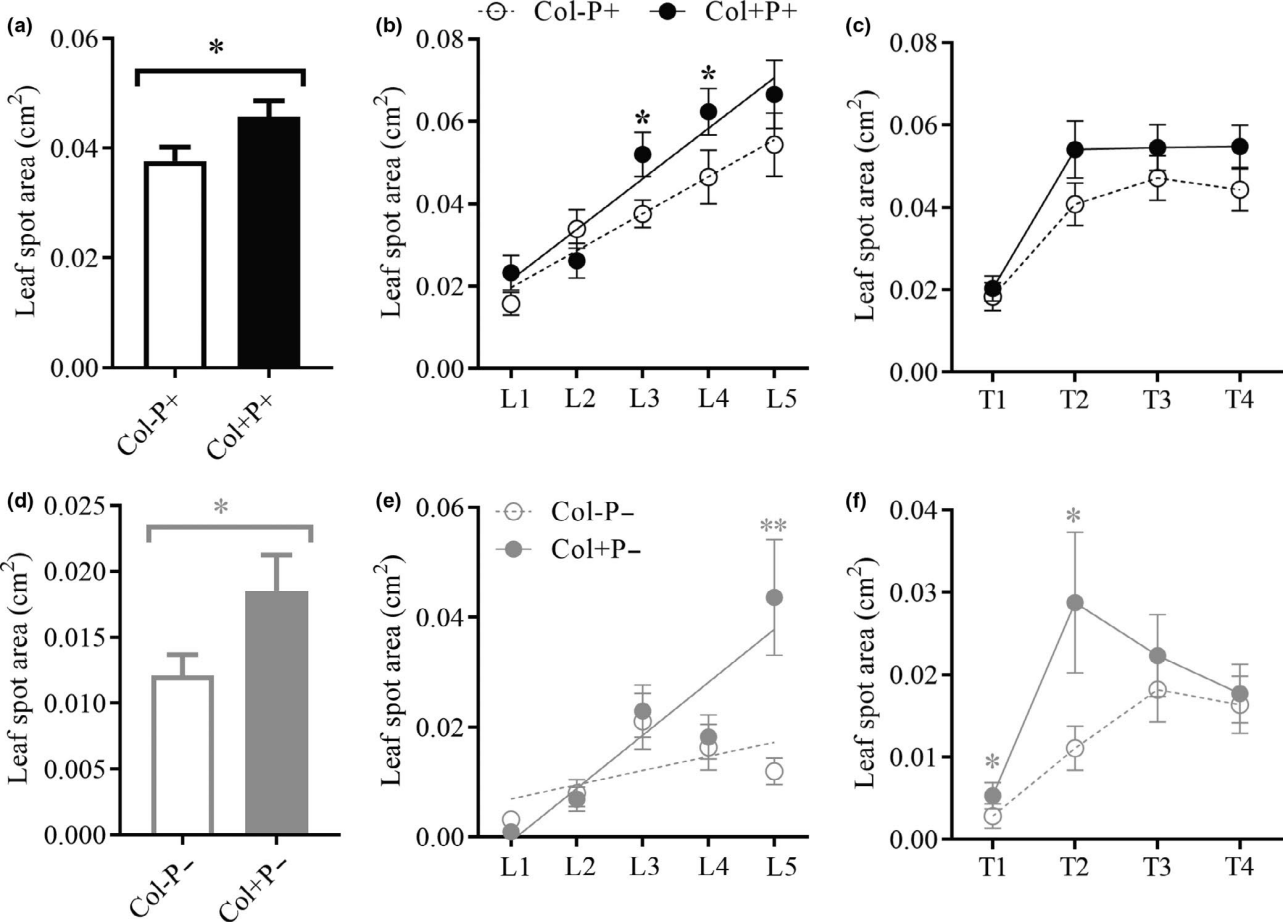
Disease development caused by the pathogen *D. helianthin* (a–c) and physical puncture (d–f) on leaves of *A. adenophora* with and without *Colletotrichum* sp. (a, d), on leaves of different ages (b, e) and at different times after pathogen inoculation or physical puncture (c, f). Col+ and Col− represent leaves with and without inoculation with *Colletotrichum* sp. P+ represents inoculation with pathogenic fungi, and P− represents physical puncture. L1‐5 represent different leaf ages (b). T1‐4 represent different durations of pathogen infection or physical puncture, with a continuous interval of 1 week (c). Nonparametric Mann–Whitney U tests were used to identify the difference in leaf spot area between leaves with and without the foliar fungus *Colletotrichum* sp. (Col− versus Col+; * represents *p* < 0.05) (a, d). The * in panel (b, e, f) indicates a significant difference in disease development between Col− and Col+ on the different leaf ages and different durations ( * represents p < 0.05; ** represents p < 0.01). Linear regression was used to analyze the relationship between the leaf spot area and leaf age (b, e), as well as with the time after physical puncturing (c, f). The error bar represent the standard error

Regardless of the leaf age or inoculation time, inoculation with the foliar fungus *Colletotrichum* sp. worsened the pathogenicity of *D. helianthi* on *A. adenophora* (Col−P + versus Col+ P+, *p* = 0.034) (Figure 5a). The leaf spot area was significantly positively correlated with leaf age ((Col−P+): *R* ^2^ = 94.78%, *p* = 0.005; (Col+ P+): *R* ^2^ = 92.13%, *p* = 0.010), and the disease developed faster with than without *Colletotrichum* sp. inoculation ((Col−P+): Slope = 0.008987 ± 0.001216; (Col+ P+): Slope = 0.01227 ± 0.002071), particularly for older leaves (*p*_3th_ = 0.036, *p*_4th_ = 0.034) (Figure 5b). Leaf spots appeared one week after inoculation, quickly developed over the following week and then remained stable (Figure 5c). In addition, the healthy Col+ leaves developed a larger leaf spot area than healthy Col− leaves only through puncturing (Col−P− versus Col+ P−, *p* = 0.023) (Figure 5d), and symptoms worsened with leaf age ((Col−P−): R^2^ = 34.31%, *p* = 0.2994; (Col+ P−): R^2^ = 85.46%, *p* = 0.0246) (Figure 5e). Similarly, the disease development was also halted two weeks after wounding (Figure 5f).

### 3.3 Effect of the foliar fungus *Colletotrichum* sp. on herbivory on *A. adenophora* leaves

**Figure 6:**
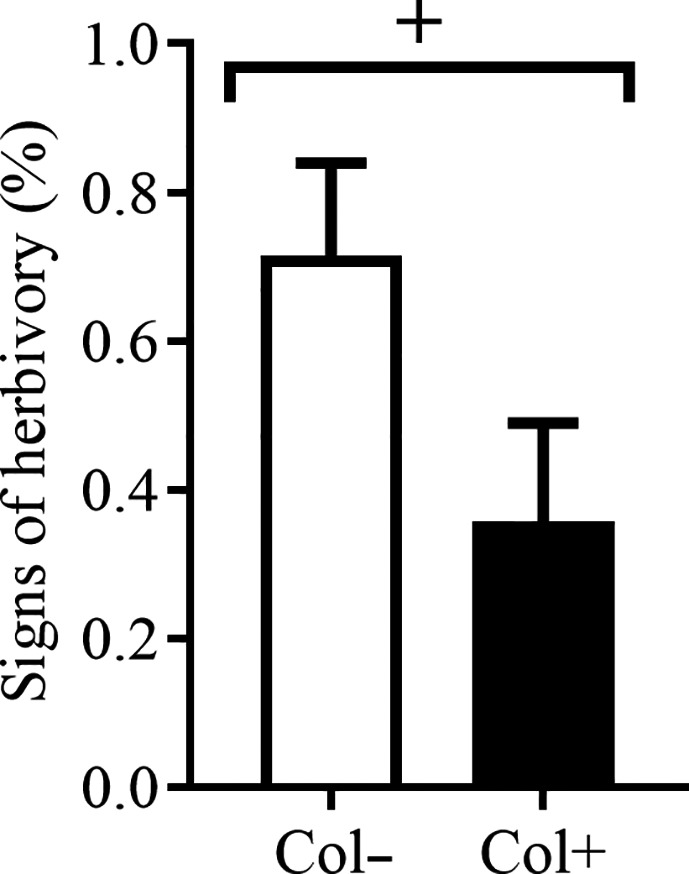
Herbivores on the leaves of *A. adenophora* with and without the foliar fungus *Colletotrichum* sp. Col+ and Col− represent leaves with and without *Colletotrichum* sp. inoculation. Nonparametric Mann–Whitney U tests were used to identify differences in herbivory between Col+ and Col− leaves (+ represents *p* < 0.1). The error bar represents the standard error

Inoculation with *Colletotrichum* sp. marginally reduced the signs of herbivory on seedlings in the wild (*p* = 0.063) (Figure 6).

## 4 DISCUSSION

Local soil biotic and abiotic factors ultimately determine the positive or negative effects of PSF on plants (Bennett & Klironomos, 2019). Our report characterized the growth response of the invasive plant *A. adenophora* when grown in agricultural and forest soils upon interaction with a foliar fungus. We found that the soil source played the most important role in the growth of *A. adenophora*. Although the forest soil showed significantly higher levels of macronutrients, such as N, P, and K, than the agricultural soil (Table 2), on average, all measurements for *A. adenophora* growth were lower in forest than in agricultural soil (Figure 4). Because the soil, as an inoculation source, was mixed with a sterilized matrix at a volume ratio of 1:9 and watered with Hoagland nutrient solution during the growth of *A. adenophora* (see Methods), the effects of the macronutrients in the soils on *A. adenophora* were likely lessened. These data thus reflected that the unmeasured micronutrients in the agricultural soil still contributed to the growth of *A. adenophora*. For example, Zhang et al. (2004) found that *A. adenophora* tends to enrich soil selenium.

Regarding the soil biota effects, we found that sterilization caused declines in most measures of *A. adenophora* growth in forest soil, including LDB per unit area, ADB and SL, and conversely, improved the LDB per unit area in agricultural soil (Figure 4). These findings suggested that *A. adenophora* experienced positive soil biota effects in forest soil but negative or neutral effects in agricultural soil, supporting previous results that soil biota from different habitats have distinct inhibitory effects on *A. adenophora* growth (Niu et al., 2007; Xiao et al., 2014). The negative feedback in agricultural soil indicates that heavy loads of pathogens exist in these soils (Ashizawa et al., 2010; Etebu & Osborn, 2010; Kohn, 1995). In contrast, the positive effect of forest soils on *A. adenophora* growth may be related to the high abundance and/or diversity of beneficial microbes, for example, AM fungi, in intact forest soils (Bordoloi et al., 2015). The invasiveness of a given plant and the invasibility of the ecosystem together determine the successful colonization of exotic plants in non‐native habitats (Milbau & Nijs, 2004). Therefore, the positive feedback in forest soil does not indicate that *A. adenophora* invades this habitat more easily than agricultural soils, which may contain unknown nutrients that support *A. adenophora* growth better than forest soils. Great variation can be observed even among agricultural soils (Franklin & Mills, 2003) based on many factors, and the same is true for forest soils (Yang et al., 2018). Therefore, a variety of factors need be ruled out before multiple soil types in both groups can be used in experiments. Nonetheless, the combined effects of both soil biota and nutrient limitation must be considered in evaluations of the invasiveness of species and the invasibility of ecosystems.

Interestingly, inoculation with the foliar fungus *Colletotrichum* sp. changed the growth response of *A. adenophora* in different soil types, for example, it decreased the aboveground biomass of *A. adenophora* in nonsterilized forest soils but had no effect on *A. adenophora* growth in agricultural soils, whether sterilized or nonsterilized (Figure 4b). Moreover, this foliar fungus mainly affected aboveground rather than belowground growth, in particular by decreasing the LDB per unit area of *A. adenophora* in both sterilized and nonsterilized forest and agricultural soils (Figure 4a). Similarly, Newcombe et al. (2009) demonstrated that the foliar fungus *Fusarium* (CID107) inhibited the development of *C. stoebe* leaves and led to a decrease in aboveground biomass. Our data indicate that a given foliar fungus can have a complex interaction with hosts and impact different biological traits of hosts depending on the soils of the habitat and the presence of soil biota.

Many studies have verified that foliar fungi can help invasive host plants resist pathogens; for example, Currie et al. (2019) demonstrated that fungal endophytes appeared to be antagonistic to rust fungus (*Puccinia komarovii*). In this study, we found that the foliar fungus *Colletotrichum* sp. promoted the pathogenicity of *D. helianthi* on *A. adenophora* leaves (Figure 5a). Moreover, when compared with leaves without *Colletotrichum* sp. inoculation, the spot area formed by *D. helianthi* on leaves inoculated with *Colletotrichum* sp. developed faster with increasing leaf age (Figure 5b). These results suggest the existence of a synergism between these two fungi that causes them to be more virulent to *A. adenophora*. Coinfection is common in nature and usually increases the pathogenicity of pathogens to the host (Laine, 2011). For example, coinfection with *Verticillium dahliae* and *Colletotrichum coccodes* causes more severe foliar disease symptoms and crown rot in potato (Nicola) than inoculation with either of the two pathogens separately (Tsror & Hazanovsky, 2001).

In addition, although *Colletotrichum* sp. JK99 has been isolated from healthy leaves and reported as an endophyte of *A. adenophora* (Mei et al., 2014), we found that healthy Col+ leaves developed a larger leaf spot area than healthy Col− leaves only through puncturing (Figure 5d), and symptoms worsened with leaf age (Figure 5e). This finding confirms that the foliar fungus *Colletotrichum* sp. is in fact a latent pathogen of *A. adenophora*, as previously reported for other invasive plant species (de Macedo et al., 2013; Newcombe et al., 2009) and supports the viewpoint that latent pathogens induce disease symptoms when plant tissues are physically damaged (Viret & Petrini, 1994). *Colletotrichum* has various life habits and can be broadly categorized as necrotrophic, hemibiotrophic, latent or quiescent and endophytic (De Silva et al., 2017). Some endophytic fungi, such as latent pathogens and quiescent pathogens, may induce disease symptoms later when the plant tissues age or become weakened (Kogel et al., 2006; Viret & Petrini, 1994). Indeed, very recently, our group indicated that many members of *Colletotrichum* sp. were asymptomatic latent pathogens of both *A. adenophora* and co‐occurring native plants (Chen et al., 2020). Nonetheless, the disease symptoms elicited by *Colletotrichum* sp. were very weak, and disease development was halted within two weeks after wounding (Figure 5f).

Foliar fungi, as a secondary metabolite source for plants, can provide their hosts with antiherbivore defenses (Nisa et al., 2015). A recent review indicates that fungal endophytes commonly have a negative effect on insect herbivores (Gange et al., 2019). Our data also showed that inoculation with *Colletotrichum* sp. marginally reduced the signs of herbivory on intact *A. adenophora* seedlings by natural insect enemies in the wild (Figure 6). The plant pathogen *Colletotrichum* sp. has previously been reported to cause extensive mortality in an insect herbivore by directly colonizing insect tissue (Graziosi & Rieske, 2015). Similarly, the members of *Colletotrichum* have been observed to infect scale insects (Marcelino et al., 2008). However, it is not known whether *Colletotrichum* species produce defense compounds to directly kill insects. Here, it was also unclear how *Colletotrichum* sp. JK99 modifies the herbivory of *A. adenophora*.

In conclusion, our preliminary study characterized the interactions among an invasive plant, a foliar fungus and natural enemies aboveground and belowground. We found that the soil type played the most important role in the growth of *A. adenophora*, and the role of the foliar fungus, *Colletotrichum* sp., varied according to the habitat but primarily adversely impacted leaf development in *A. adenophora*. The foliar fungus *Colletotrichum* sp. might be a latent pathogen, and it enhanced the pathogenicity of *D. helianthi* on *A. adenophora* leaves; this fungus also marginally reduced the signs of herbivory on seedlings from natural insect enemies in the wild. Previously, invasive hosts have been shown to benefit from the presence of foliar fungi (Aschehoug et al., 2012; Currie et al., 2019; Evans, 2008; Xiao et al., 2012). The adverse impacts in this study may be related to the latent pathogenic effects of this foliar fungus but also reflect the fact that the ecological functions of foliar fungi are usually context dependent (Hawkes & Connor, 2017). In particular, our study highlights the need to consider both aboveground and belowground biota in different habitats when evaluating the effects of foliar fungi.

## CONFLICT OF INTEREST

The authors declare no competing financial interests.

## AUTHOR CONTRIBUTION

**Kai Fang:** Data curation (lead); Formal analysis (lead); Writing‐original draft (lead). **Li‐Min Chen:** Data curation (equal); Investigation (lead); Methodology (lead); Resources (lead). **Han‐Bo Zhang:** Conceptualization (lead); Funding acquisition (lead); Supervision (lead); Writing‐review & editing (lead).

## Supporting information

Fig S1‐S5

Note S1

Table S1

## Data Availability

All raw data used in this manuscript have been deposited in the Dryad Digital Repository: https://doi.org/10.5061/dryad.fttdz08r6
