# Supplementary material for: Evaluation of foliar fungus‐mediated interactions with below and aboveground enemies of the invasive plant Ageratina adenophora
Source: Ecol Evol. 2020 Nov 25;11(1):526–35. doi: 10.1002/ece3.7072 (PMC7790651; doi:10.1002/ece3.7072)
Supplement: Supplementary file 1 — Fig S1‐S5 [file ECE3-11-526-s001.docx]

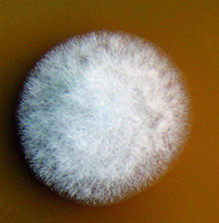


**Figure S1** Colony morphology of *Colletotrichum* sp. on MEA medium.


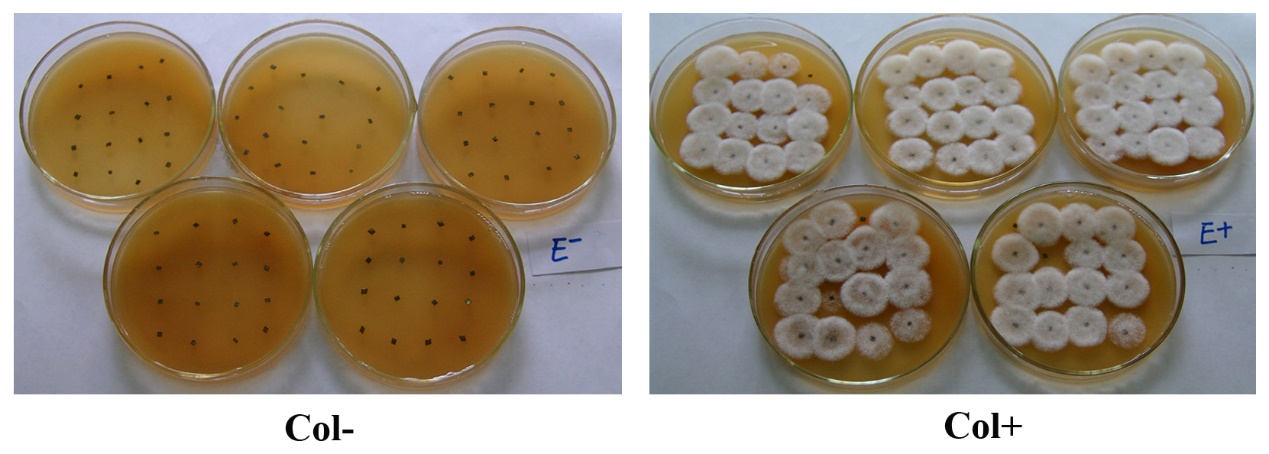


**Figure S2** Detection of foliar fungus *Colletotrichum* sp. in leaves by MEA medium. The left panel was the non-inoculated leaves (Col-, control plants), while the right panel was the inoculated leaves of *Colletotrichum* sp. (Col+).


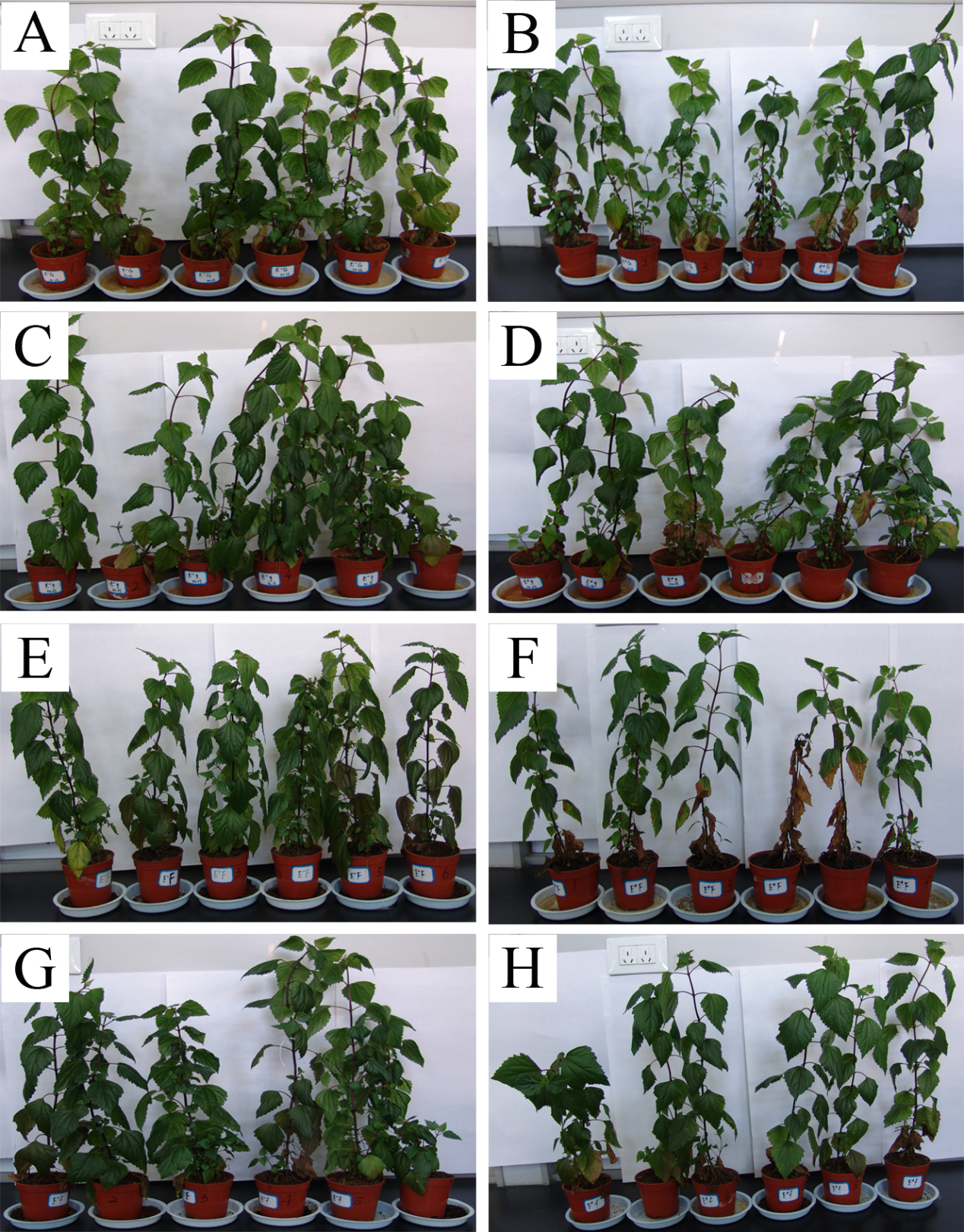


**Figure S3** Effects of inoculation or non-inoculation of *Colletotrichum* sp. on the growth performance of *A. adenophora* in agricultural (A, B, C and D) and forest soils (E, F, G and H). The seedlings of B, D, F and H were inoculated with *Colletotrichum* sp., while the seedlings of A, C, E and G were not. The soil of A, B, E and F were not sterilized, while the soil of C, D, G and H were sterilized.


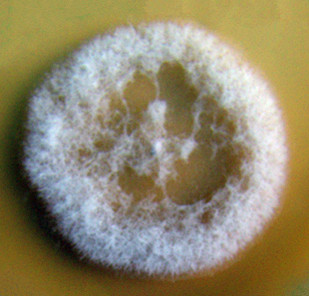


**Figure S4** Colony morphology of *Diaporthe helianthi* on MEA medium.


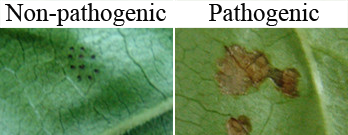


**Figure S5** Comparison of leaf spots of *A. adenophora* caused by sterilized ager (left) and pathogen *Diaporthe helianthi* (right).
