# Supplementary material for: Evaluation of foliar fungus‐mediated interactions with below and aboveground enemies of the invasive plant Ageratina adenophora
Source: Ecol Evol. 2020 Nov 25;11(1):526–35. doi: 10.1002/ece3.7072 (PMC7790651; doi:10.1002/ece3.7072)
Supplement: Supplementary file 2 — Note S1 [file ECE3-11-526-s002.docx]

1. Data of growth experiment of *Ageratina adenophora* under different soil types was included the sheet of LDB per unit area, ADB, BDB and SL.

2. Data of inoculation experiment of leaf pathogenic fungi was included the sheet of leaf spot area.

3. Data of herbivory experiment was included the sheet of signs of herbivory.
